# Patients’ preferences for postmenopausal hormone receptor-positive, human epidermal growth factor receptor 2-negative advanced breast cancer treatments in Japan

**DOI:** 10.1007/s12282-019-00965-4

**Published:** 2019-04-04

**Authors:** Yukie Omori, Sotaro Enatsu, Zhihong Cai, Hiroshi Ishiguro

**Affiliations:** 1 Eli Lilly Japan K.K., Medicines Development Unit Japan, Akasaka Garden City 13F, 4-15-1, Akasaka, Minato-ku, Tokyo, 107-0052 Japan; 2 Eli Lilly Japan K.K., Medicines Development Unit Japan, 5-1-28, Isogamidori, Chuo-ku, Kobe, Japan; 3 International University of Health and Welfare Hospital, Nasushiobara, Japan

**Keywords:** Breast cancer, Patient preference, Cyclin-dependent kinase 4, Cyclin-dependent kinase 6

## Abstract

**Background:**

This study aimed to identify factors affecting patients’ preferences for postmenopausal hormone receptor-positive (HR+), human epidermal growth factor receptor 2-negative (HER2−) advanced breast cancer treatments, their relative importance, and impact of sociodemographic/clinical characteristics.

**Methods:**

Japanese postmenopausal patients with HR+ breast cancer chose between 2 hypothetical treatments for HR+/HER2− advanced breast cancer using an online discrete choice experiment, defined by different levels of 5 attributes: progression-free survival (PFS), incidence of diarrhea (IOD), frequency of loose stools of grade 1–3 severity (FOS), duration of diarrhea (DOD), and route/frequency of administration (RFA). Conditional logit modeling identified relative preferences for each attribute. Subgroup analyses, based on sociodemographic characteristics (age, employment status, age of youngest child, marital status) and clinical characteristics (relapse/metastasis, hormone sensitivity), identified factors affecting preferences.

**Results:**

Of 896 participants screened, 258 eligible participants were included in analyses. Patient preferences, when the potential frequency of diarrhea was grade 2, were (strongest to weakest): PFS, DOD, FOS, IOD, RFA; however, when the potential frequency of diarrhea was grade 3, FOS became most important. Sociodemographic/clinical characteristics tended to affect preferences.

**Conclusions:**

Japanese postmenopausal patients with HR+ breast cancer preferred treatments that extend PFS despite potential grade 2 diarrhea. However, when diarrhea severity increased to grade 3, patients were more willing to sacrifice PFS to avoid more frequent diarrhea. Prevention or limitation of diarrhea to grade ≤ 2 is important for maintaining patients’ motivation for treatment that can extend PFS. Additionally, patient characteristics (age, family context, therapeutic experience) should be considered during treatment choice.

**Electronic supplementary material:**

The online version of this article (10.1007/s12282-019-00965-4) contains supplementary material, which is available to authorized users.

## Introduction

GLOBOCAN 2018 estimates that over 2 million individuals globally were affected by breast cancer, making it the second most common cancer worldwide in 2018 [1]. In Japan, approximately 66,000 new cases of breast cancer were reported in 2018 [2]. An increased trend in the morbidity and mortality of breast cancer since the 1970s has been observed, and it is estimated that the average annual incidence of breast cancer, in combination with the increasing number of patients aged 80 years, will increase until 2025 [3, 4]. Hormone receptor-positive (HR+) breast cancer accounts for approximately 80% of all breast cancer cases [5] and single-agent endocrine therapy is the mainstay for treatment for HR+, human epidermal growth factor receptor 2-negative (HER2−) advanced breast cancer (e.g., fulvestrant, a selective estrogen receptor). However, resistance to endocrine therapy frequently develops and relapse may occur in almost all patients [6]. Clinical research has focused on enhancing and improving outcomes of endocrine-based therapy to augment disease control, delay the use of chemotherapy, and optimize the length and patients’ quality of life [7].

Currently, the focus of research has shifted to investigating combinations of targeted agents and endocrine therapy in patients with advanced breast cancer [8]. For example, the mammalian target of rapamycin (mTOR) inhibitor everolimus in combination with exemestane has demonstrated benefit in patients with HR+ advanced breast cancer [9]. Similarly, cyclin-dependent kinase (CDK) 4 and 6 inhibitors in combination with hormonal agents have also demonstrated efficacy in treating breast cancer [10, 11]. While treatment efficacy has been improved by the development of new drugs, various treatment-related adverse events have been reported. Debilitating adverse events, such as nausea, vomiting, diarrhea, and sensory neuropathy can negatively affect patients’ quality of life, and therefore, their impact on patient decision-making should not be underestimated [12].

With the increasing number of treatment options, patients’ preferences may play an important role in the selection of breast cancer treatments. Unfortunately, physicians often fail to understand patients’ perspectives and preferences in their treatment decision-making [13, 14]. It is important to identify patient preferences for treatment and the factors affecting their preferences, as well as to understand how patients weigh treatment efficacy and regimen against the potential toxicity of different treatments. These findings could potentially enhance the treatment choice process and allow physicians to tailor treatment to an individual patient.

Abemaciclib is a CDK4 and 6 selective oral inhibitor that induces prolonged cell cycle arrest and subsequent cellular senescence or apoptosis [15]. Recent results from a phase III trial comparing the safety and efficacy of abemaciclib plus fulvestrant versus placebo plus fulvestrant in women with HR+/HER2− advanced breast cancer whose disease progressed while receiving endocrine therapy (MONARCH 2) showed that abemaciclib in combination with fulvestrant significantly prolonged median progression-free survival (PFS) by 7 months and had a tolerable safety profile [16]. In this trial, diarrhea was reported as the main significant adverse event (AE) related to abemaciclib. Of 441 patients who received abemaciclib plus fulvestrant, 381 patients (86.4%) experienced diarrhea (322 [73.0%] with grades 1 and 2; 59 [13.4%] with grade 3; and no patients with grade 4; by the grading of Common Terminology Criteria for Adverse Events [CTCAE] v4.0) [16]. Therefore, we wanted to assess the trade-offs that patients with breast cancer were willing to make for treatments according to efficacy, toxicity, and convenience related to these regimens.

The primary objective of this study, conducted in postmenopausal women with HR+ breast cancer in Japan, was to identify the treatment attributes and their relative importance to patients’ treatment preference using a discrete choice experiment (DCE) online survey. The second objective of our study was to explore whether patients’ sociodemographic and clinical characteristics would affect their preference in their choice of treatment.

## Methods

### Study design

This was a DCE to identify the factors affecting patient preferences for drug treatment and their relative importance in postmenopausal patients with HR+ breast cancer in Japan. The participants completed the internet-based survey at home. The survey comprised a sociodemographic and clinical information questionnaire, which was followed by DCE questions. Screening was conducted to ensure the participants’ eligibility according to inclusion and exclusion criteria. A pilot study was conducted to estimate the number of valid responses required in the main study and to determine whether participants could comprehend the questionnaire. Based on the pilot study findings, a premise that explained the characteristics of each attribute was added to the questionnaire used in the main study. No major modifications to the study design or questionnaire were necessary. The screening and the pilot study were conducted in January 2018, while the main study was conducted in February 2018. The study protocol was approved by an independent ethical review board (Non-profit organization MINS Institutional Review Board, Tokyo, Japan) and the study was conducted in compliance with Good Pharmacoepidemiology Practices, applicable laws, regulations of Japan, and the 1964 Declaration of Helsinki and all subsequent revisions. All participants provided their online informed consent before answering the questionnaire.

### Study population

Potential participants were identified from a web panel owned by INTAGE Inc., which included approximately 1400 patients who had received any treatments within the past year and 1100 patients who were receiving any treatments in Japan as of August 2016. They were invited via e-mail to participate in the study, and those who agreed to participate and answered the screening questionnaire were assessed for their eligibility according to the inclusion and exclusion criteria. It would have been ideal to include the same population studied in the MONARCH 2 trial (i.e., women aged ≥ 18 years with HR+/HER2− breast cancer and with pre- or perimenopausal status who received a gonadotropin-releasing hormone agonist) [16]. We were, however, constrained by the population registered in the INTAGE Inc. panel, and only postmenopausal women aged ≥ 45 years with HR+ breast cancer were included in this study. Given the small number of patients with HER2− disease registered in the panel, HER status was not a study inclusion or exclusion criterion. In addition, this study involved only postmenopausal women for clarification of eligibility. Participants were excluded from the study if they were pre-menopausal, had difficulty comprehending the questionnaire (determined by their response to a fixed-choice question), or withdrew informed consent.

### DCE content

#### Questionnaire structure

The questionnaire comprised three sections: sociodemographic questions (age, gender, highest educational level, employment status, marital status, and age of youngest child); clinical characteristics (disease duration, stage at the initial diagnosis, prior therapies, hormone sensitivity, experience of relapse/metastasis, and menstrual status); and preferences for treatment based on treatment attributes such as efficacy, safety, and route and frequency of administration. Hormone sensitivity of respondents who had experienced a relapse or metastases was classified as no experience of endocrine therapy, primary resistance, acquired resistance, and hormone responsive by their answers to specific questions according to the 2nd International Consensus Conference for advanced breast cancer (ABC2) guidelines [7]. Some answers included “do not want to answer” or “others” to reduce respondents’ burden and minimize withdrawal prior to completion of the questionnaire.

#### Attributes and levels

Findings from the MONARCH 2 trial guided selection of the appropriate attributes and their levels [16]. Five attributes were selected for the DCE (Table 1). Attributes included PFS; frequency of stools (FOS), which represented the increase above average in the number of loose stools per day; incidence of diarrhea (IOD); duration of diarrhea (DOD); and route and frequency of administration (RFA) of the treatment. Diarrhea was selected as a key side effect attribute because it is one of the most frequently reported AEs in clinical trials of abemaciclib [16] and is considered the most concerning AE associated with abemaciclib-containing regimens for physicians. The levels of FOS were ranked based on the grading of diarrhea by CTCAE v4.0. The levels of RFA were based on the 2 regimens compared in MONARCH 2 trial (i.e., abemaciclib plus fulvestrant and fulvestrant-only regimen). Attributes and their levels were described in lay terminology to increase respondents’ understanding and, hence, to augment the response rate (Table 1).

**Table 1:** Attributes and levels used in the discrete choice experiment

Attribute	Level
Progression-free survival (PFS)	1. Approximately twice as long as existing treatment (16 months) 2. Approximately the same as existing treatment (9 months)
Frequency of stools^a^ (FOS)	1. Increase of 3 stools per day over baseline 2. Increase of 6 stools per day over baseline 3. Increase of 9 stools per day over baseline
Incidence of diarrhea (IOD)	1. 9 of 10 patients will experience diarrhea 2. 2 of 10 patients will experience diarrhea
Duration of diarrhea (DOD)	1. 2 weeks 2. 2 months
Route and frequency of administration (RFA)^b,c^	1. Oral administration (twice a day) + intramuscular injection (once every 4 weeks) 2. Intramuscular injection only (once every 4 weeks)

^a^Refers to grading of diarrhea in Common Terminology Criteria for Adverse Events v4.0: grade 1, increase of < 4 stools per day over baseline; grade 2, increase of 4–6 stools per day over baseline; grade 3, increase of ≥ 7 stools per day over baseline

^b^Intramuscular injection (once every 4 weeks) refers to administration of fulvestrant in the MONARCH 2 trial

^c^Oral administration (twice a day) refers to administration of abemaciclib in the MONARCH 2 trial

#### Choice sets

Alternatives were created by choosing a level from each attribute. The alternatives shown to each respondent were theoretically 48 (2^4^ × 3) in total in a full factorial design for this study but were limited to 8 alternatives according to orthogonal array to reduce respondents’ burden while completing the questionnaire. Choice sets about hypothetical treatments were created using a shifting method [17]. Each questionnaire was composed of 8 choice sets and each choice set contained 5 attributes with different levels (Supplementary Table 1). A fixed-choice question composed of one clearly preferable option and one clearly non-preferable option were included in the questionnaire to verify respondents’ understanding of the DCE and to check the validity and internal consistency of the DCE design. The respondents who did not choose the preferable response option to the question were excluded from the analysis. The order of choice sets was randomly changed for each respondent. Respondents were asked to choose one preferred treatment alternative in each choice set (Supplementary Table 1). Treatments were labeled as Treatment A and Treatment B; no product names were presented in the survey. Characteristics of each attribute in 2 hypothetical treatments shown as choices in the experiment were presented to the respondents as a premise of the DCE.

### Statistical analysis

The sample size was estimated using DCE analysis parameters, which included 8 choice pairs, 2 alternatives per choice pair, and a maximum of 3 levels for any individual attribute. Based on these study parameters and practical guidelines for robust quantitative research study, a sample size of 300 patients was targeted for this study [18]. A pilot study was conducted to identify the expected number of valid responses of respondents who matched the inclusion or exclusion criteria. A total of 39 respondents participated in the pilot study and 36 valid responses were collected. Based on the pilot study result, a sample size of between 100 and 300 participants was considered reasonable and selected as the final sample size. Data from the pilot study were not included in this analysis.

The primary analysis identified the preference weights for each level of each study attribute using a conditional logit model. The preference weights were evaluated with *β* coefficient and standard error. In addition, a conditional logit model including patient-specific covariates, such as patient sociodemographic characteristics [i.e., age (45–59 years or ≥ 60 years); employment status (employment or no employment); age of youngest child (no children, children aged ≥ 20 years, and/or children aged < 20 years); marital status (single, married, or divorced/separated/widowed)]; clinical characteristics [experience of relapse or metastasis (yes or no); and hormone sensitivity status (hormone responsive, primary/acquired resistance or no experience of endocrine therapy)], was used to identify factors that affect patient preferences in choice of treatment. Further, subgroup analyses by patient sociodemographic and clinical characteristics were conducted. All statistical tests were two-sided and used a significance level of 0.05 unless otherwise noted. No adjustments for multiple comparisons were performed. Missing data were not imputed. Statistical analyses were performed using STATA/IC 15.1 (StataCorp LLC, TX, USA).

## Results

### Respondents’ disposition, sociodemographic, and clinical characteristics

**Fig. 1:**
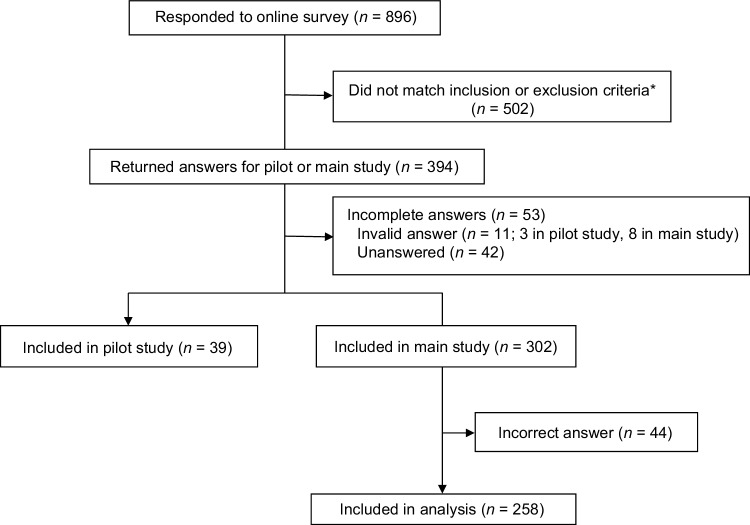
Patient disposition. *Some patients met more than one criterion. *HR* hormone receptor

Among all potential participants who were emailed an invitation to participate, 896 agreed to participate in the study and answered the screening questionnaire. Of these, 394 participants matched the inclusion or exclusion criteria. Of these eligible participants, only 341 who had complete answers entered the pilot study (*n* = 39) or the main study (*n* = 302). In the main study, 44 respondents were excluded from the analysis because they did not choose the preferable option in the fixed-choice question, thus 258 respondents with valid responses were included in the main study analysis (Fig. 1). The mean age (SD) of the respondents was 56.7 (6.7) years; >50% had attained college/university-level education; 47.7% had paid employment; 67.1% were married; 64.0% had children; with 14.3% having youngest children aged < 20 years (Table 2). Mean duration since diagnosis was 6.3 years; 91.5% had prior therapy with anticancer drug; 15.9% had experienced relapse/metastasis, and 84.5% were hormone responsive (Table 2).

**Table 2:** Self-reported sociodemographic and clinical characteristics of respondents included in the analysis

Characteristic	All (*n* = 258)
Age	
Mean ± SD, years	56.7 ± 6.7
45–59 years, *n* (%)	188 (72.9)
≥ 60 years, *n* (%)	70 (27.1)
Level of highest education, *n* (%)^a^	
Junior high school (year 9)	2 (0.8)
High school (year 12)	75 (29.1)
Vocational school	30 (11.6)
College/university	146 (56.6)
Postgraduate	4 (1.6)
Other	1 (0.4)
Employment status, *n* (%)	
Employment	124 (48.1)
Full-time	55 (21.3)
Part-time	53 (20.5)
Self-employed	7 (2.7)
Contract employee	8 (3.1)
Student	1 (0.4)
No employment	134 (51.9)
Disemployed	20 (7.8)
Retired	10 (3.9)
Housewife	90 (34.9)
Other	14 (5.4)
Marital status, *n* (%)	
Single	48 (18.6)
Married	173 (67.1)
Divorced/separated/widowed	36 (14.0)
Presence of children	
Yes, *n* (%)	165 (64.0)
Age of youngest child, mean ± SD, years	26.2 ± 8.2
< 20 years, *n* (%)	37 (14.3)
≥ 20 years, *n* (%)	128 (49.6)
No, *n* (%)	93 (36.0)
Disease duration, mean ± SD, years	6.3 ± 5.0
Stage at initial diagnosis, *n* (%)^a^	
0	11 (4.3)
I	106 (41.1)
II	93 (36.0)
III	20 (7.8)
IV	9 (3.5)
Unknown	19 (7.4)
Prior therapies^b^, *n* (%)	
Surgery	251 (97.3)
Radiation	179 (69.4)
Anticancer drug therapy	236 (91.5)
Other	21 (8.1)
Experience of relapse or metastasis, *n* (%)	
Yes	41 (15.9)
No	217 (84.1)
Hormone sensitivity, *n* (%)	
No experience of endocrine therapy	8 (3.1)
Primary resistance	16 (6.2)
Acquired resistance	16 (6.2)
Hormone responsive	218 (84.5)

*SD* standard deviation

^a^Due to rounding, the sum of percentages is greater than 100%

^b^Multiple answers were allowed

### Preferences for selected attributes of treatment for HR+/HER2− advanced breast cancer

**Fig. 2:**
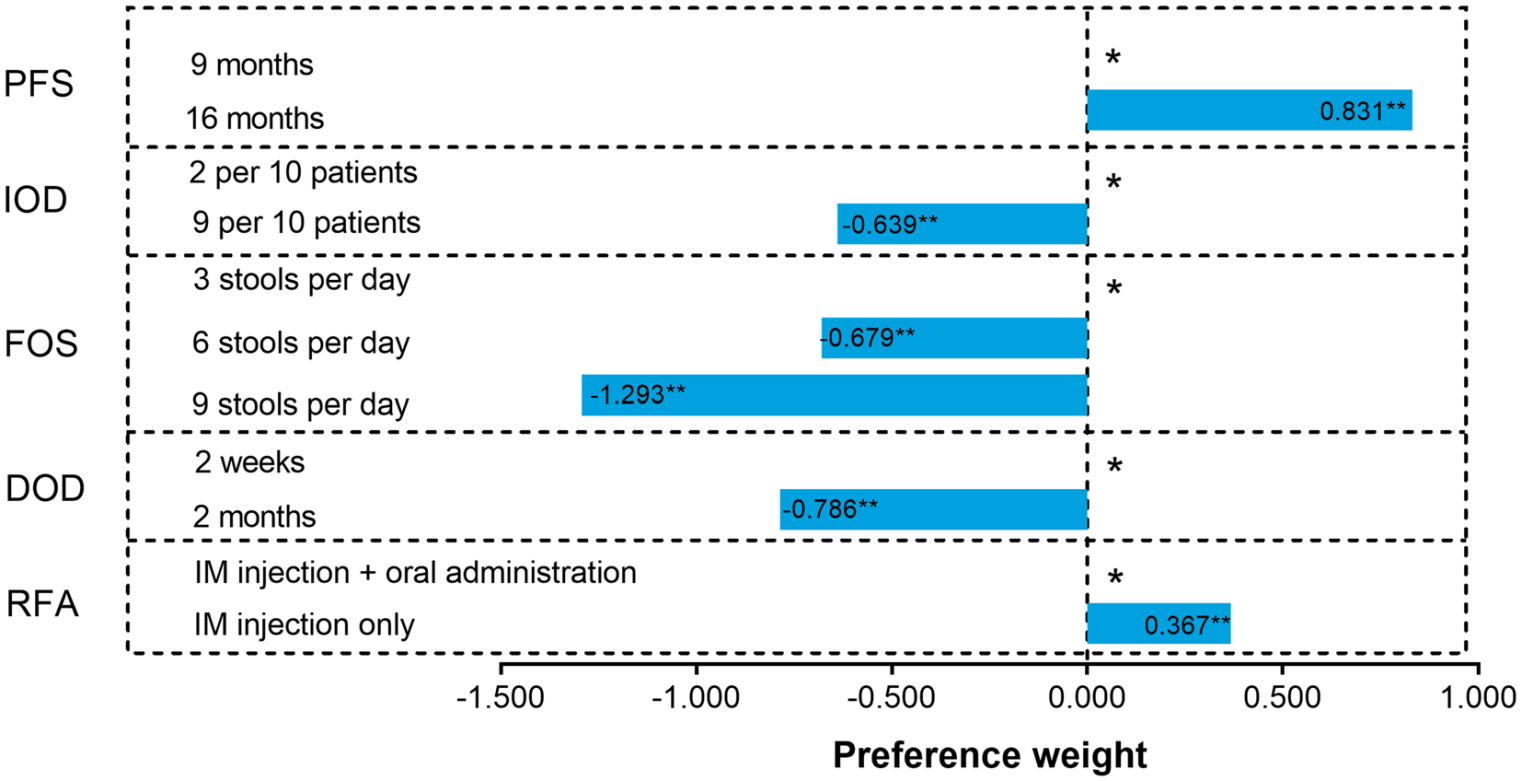
Preference weights of attribute levels in the overall sample. *As reference group. **P < 0.0001. *DOD* duration of diarrhea, *FOS* frequency of stools, *IM* intramuscular, *IOD* incidence of diarrhea, *PFS* progression-free survival, *RFA* route and frequency of administration

When presented with different options, respondents with HR+ breast cancer reported a strong preference for a treatment that can extend PFS even with the potentiality of grade 2 diarrhea. Specifically, according to the absolute magnitude of coefficients, when the FOS was 6 (grade 2 diarrhea), the order of attributes’ relative importance was the following: PFS, DOD, FOS, IOD, and RFA (Fig. 2). However, when the FOS was 9 (grade 3 diarrhea), FOS was the most important attribute for respondents (Fig. 2). All tested attributes were statistically significant (*P* < 0.0001) for their preference in choice of treatment.

### Sociodemographic and clinical characteristics and patients’ preferences

**Fig. 3:**
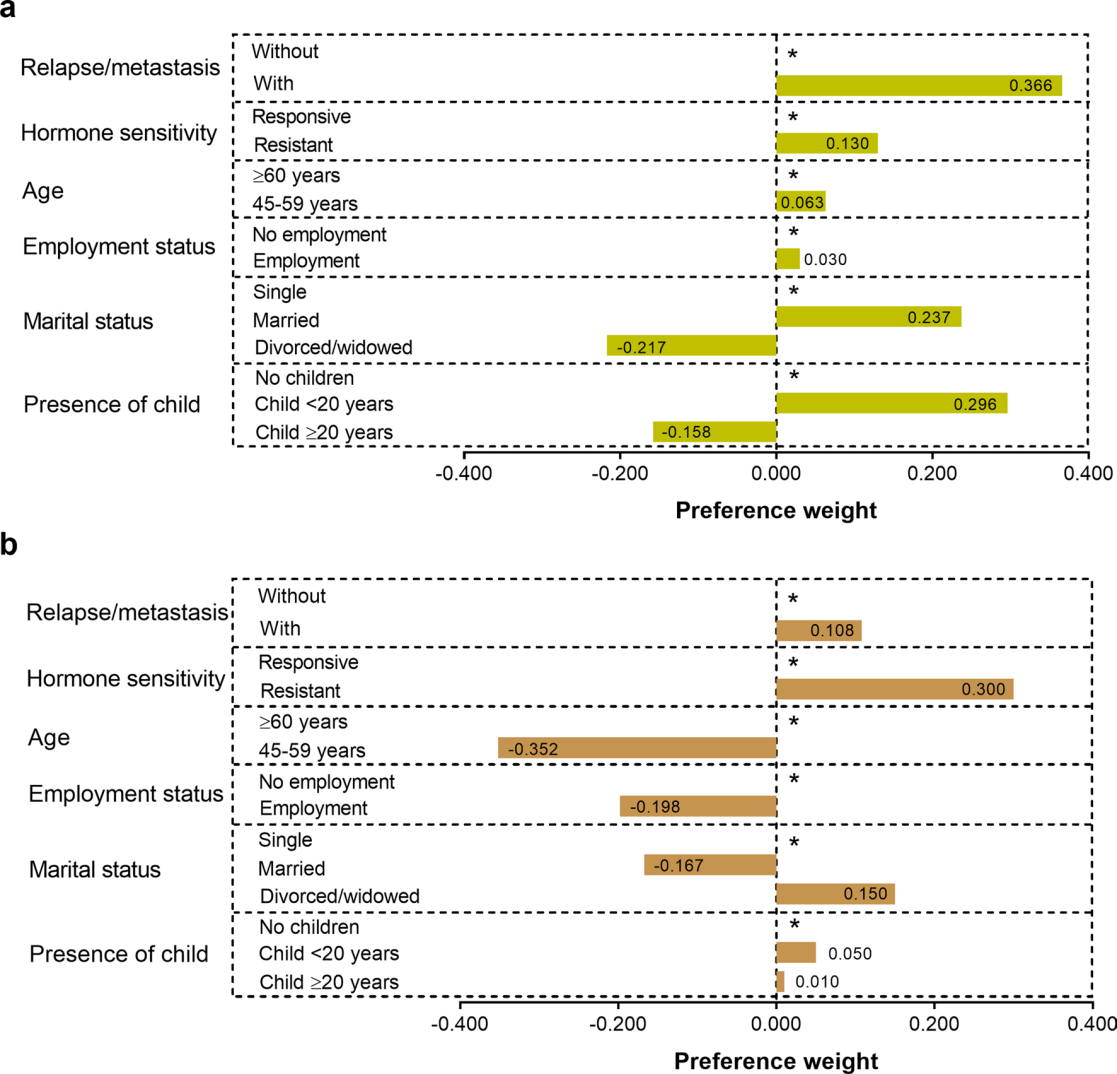
Preference weights on **a** 16-month PFS or **b** FOS of 9 (grade 3 diarrhea) when sociodemographic and clinical characteristics of respondents were included in the model. *As reference group. *PFS* progression-free survival, *Responsive* HR responsive, *Resist* primary resistance, acquired resistance, or no experience of HR therapy

Although not statistically significant, when patient-specific covariates were included in the model, the respondents who had experienced a relapse or metastases (41/258 [15.9%]) showed the strongest preference for the longest PFS (i.e., 16 months) (Fig. 3a; Table 2) and the respondents aged between 45 and 59 years (188/258 [72.9%]) showed the weakest preference for the highest FOS (grade 3 diarrhea) (Fig. 3b; Table 2).

**Fig. 4:**
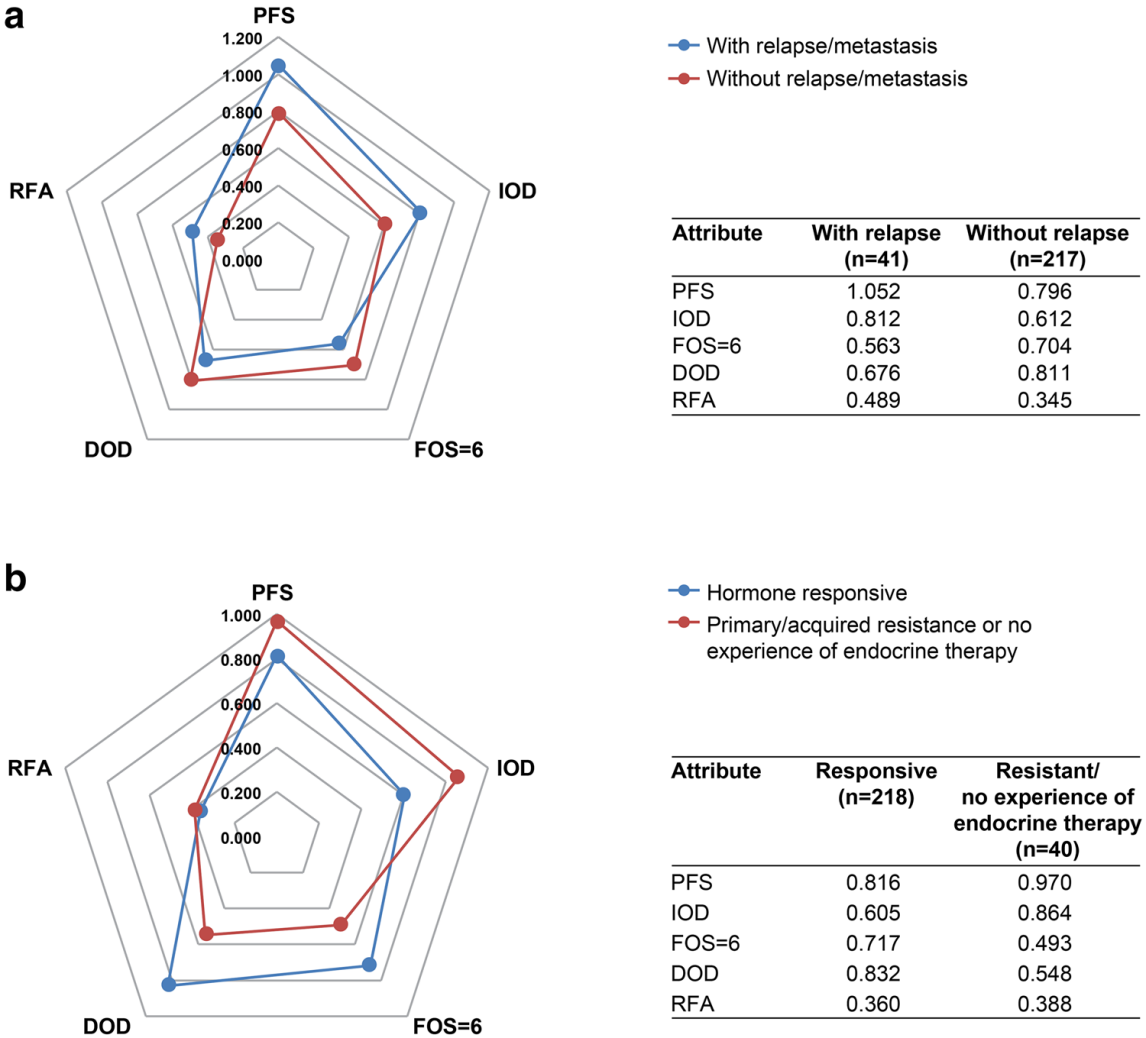
Absolute magnitude of coefficients when FOS was 6 (grade 2 diarrhea) by subgroup of **a** experience of relapse/metastasis status; **b** hormone sensitivity status. *DOD* duration of diarrhea, *FOS* frequency of stools, *HR* hormone responsive, *IOD* incidence of diarrhea, *PFS* progression-free survival, *Responsive* HR responsive, *Resist* primary resistance, acquired resistance, or no experience of endocrine therapy, *RFA* route and frequency of administration

Based on the subgroup analyses, we estimated that the clinical characteristics of patients (e.g., advanced disease, hormone responsiveness) may affect their preferences for treatment. Therefore, we explored the absolute magnitude of coefficients for each attribute by respondents’ experience of relapse/metastasis and their hormone sensitivity status. For respondents who had experience with relapse and/or metastasis (41/258 [15.9%]), when the FOS was 6 (grade 2 diarrhea), the longest PFS (16 months) was the most important attribute, but not for respondents who had no experience of relapse and/or metastasis (217/258 [84.1%]) (Fig. 4a; Table 2). The collected data were also analyzed by hormone sensitivity status, that is, hormone responsive (218/258 [84.5%]), hormone resistance (primary resistance [16/258 {6.2%}], acquired resistance [16/258 {6.2%}]), or no experience of endocrine therapy (8/258 [3.1%]). When the FOS was 6 (grade 2 diarrhea), the longest PFS (16 months) was the most important attribute for respondents who had hormone resistance or no experience of endocrine therapy, but not for patients who were hormone responsive (Fig. 4b; Table 2).

**Fig. 5:**
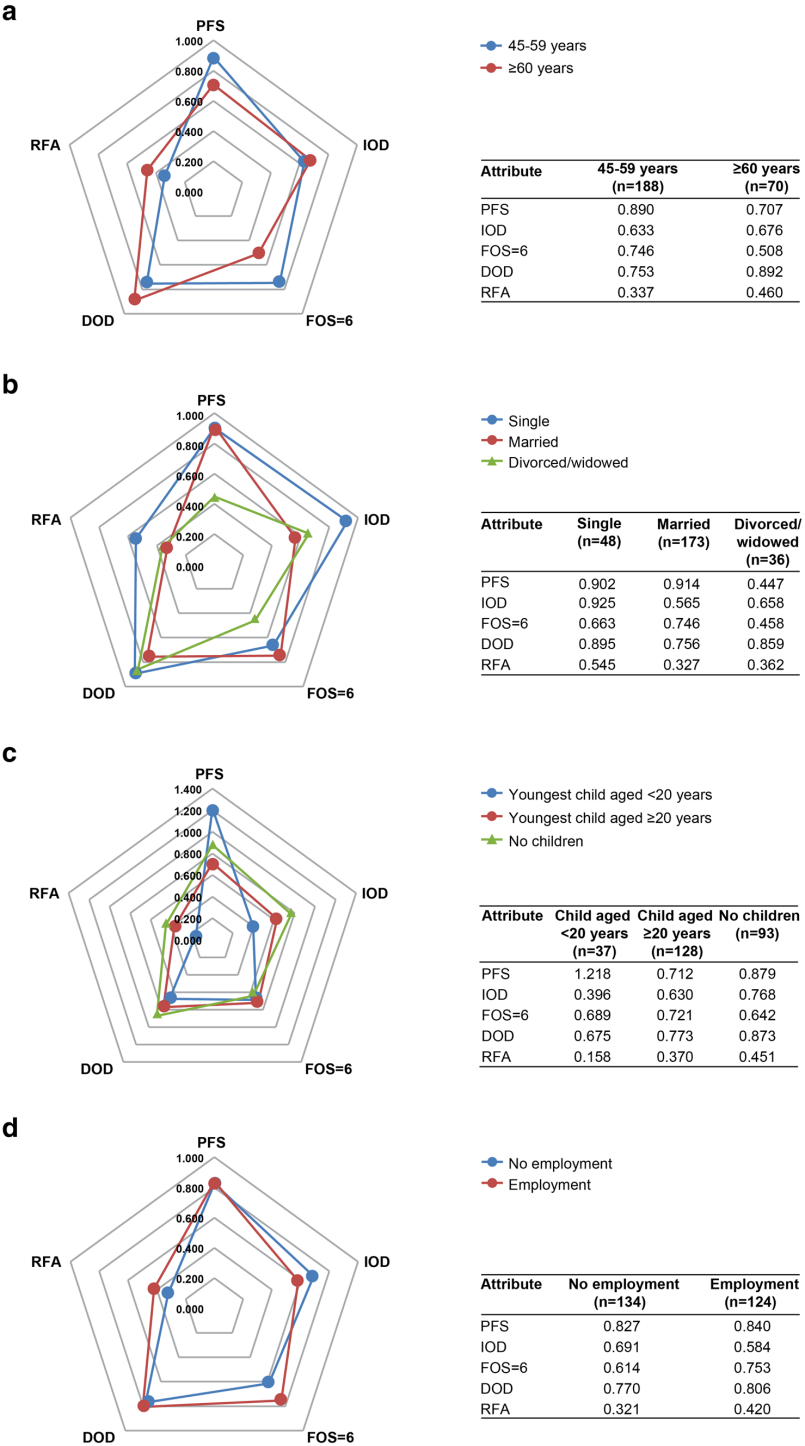
Absolute magnitude of coefficients when FOS was 6 (grade 2 diarrhea) by subgroup of **a** age; **b** marital status; **c** age of youngest child; and **d** employment status. *DOD* duration of diarrhea, *FOS* frequency of stools, *IOD* incidence of diarrhea, *PFS* progression-free survival, *RFA* route and frequency of administration

Similarly, to investigate whether respondents’ sociodemographic characteristics affect their preferences for treatment, we explored the absolute magnitude of coefficients for each attribute by respondents’ age, employment status, marital status, and the age of their youngest child. The majority of respondents (188/258 [72.9%]) were aged between 45 and 59 years (Table 2); and when the FOS was 6 (grade 2 diarrhea), these respondents considered the longest PFS (16 months) as the most important attribute, whereas the ≥ 60-year-old respondents group (70/258 [27.1%]) did not (Fig. 5a). For other characteristics, when the FOS was 6 (grade 2 diarrhea), the respondents with different marital status and child status showed different preference weights for the longest PFS. The longest PFS was the most important attribute for married respondents (173/258 [67.1%]) (Fig. 5b) and for respondents whose youngest child was aged < 20 years (37/258 [14.3%]) and for respondents who had no children (93/258 [36.0%]) (Fig. 5c). In contrast, regardless of employment status, respondents showed the strongest preference for the longest PFS (Fig. 5d).

## Discussion

This is the first DCE study to assess patients’ preferences for treatments with attributes associated with abemaciclib plus fulvestrant or fulvestrant-only treatment for postmenopausal HR+/HER2− advanced breast cancer. Postmenopausal patients with HR+ breast cancer in Japan had a strong preference for treatment that can extend PFS even with the potentiality of grade 2 diarrhea. The results also demonstrated that if the frequency of loose stools increased to grade 3, this attribute became the most important attribute in patients’ treatment preferences. Moreover, we found that patients’ sociodemographic and clinical characteristics tend to affect patients’ treatment choices. Therefore, it is important to consider patient characteristics (i.e., age, marital status, presence of dependent children, experience of relapse/metastasis, and hormone sensitivity status) when choosing treatments for advanced breast cancer.

In this study, extension of PFS was the most important attribute for postmenopausal patients with HR+ breast cancer when choosing a treatment for HR+/HER2− advanced breast cancer, despite the potential for experiencing grade 2 diarrhea. The results of this study are consistent with that of cross-sectional studies on patients’ preferences for chemotherapies used in breast cancer, which showed that survival was the most important attribute [12, 19]. However, our study showed that when diarrhea events had a greater frequency with the potential of grade 3 severity, respondents were more willing to sacrifice PFS to avoid experiencing more frequent loose stools. In MONARCH 2, most patients (70.1%) in the abemaciclib plus fulvestrant arm experiencing diarrhea did not require treatment modification (i.e., dose interruption, reduction, or discontinuation) [16]. In most cases, diarrhea was effectively managed using antidiarrheal medications and with dose adjustments; with only 2.9% of patients discontinuing study drug because of diarrhea [16]. This would suggest that effective prevention and management of diarrhea (i.e., limiting diarrhea to grade 2 and below) may limit the impact of diarrhea, and therefore, would maintain patients’ motivation for treatments that could extend PFS.

Sociodemographics or clinical characteristics of individual patients may be predictive of patient preferences [20, 21]. Experience of disease progression and treatment failure may drive patients’ preference for treatment that can prevent or delay their disease progression. Our study showed that patients who were not responsive to endocrine therapy at the time of this study because of the acquisition of resistance to endocrine therapy or patients who had no experience with endocrine therapy, and/or the experience of metastasis/relapse may affect patients’ preference for treatment in postmenopausal patients with HR+ breast cancer in Japan. A cross-sectional study showed that survival was important for women receiving chemotherapy for breast cancer regardless of whether they had early- or advanced-stage disease [12]. Furthermore, patients’ age, marital status, presence of children and their age influenced patients’ preferences for treatment in our study. Indeed, older women may be less willing to sacrifice quality of life for survival prolongation as they have different priorities from younger women [22]. Similarly, another study reported that patients with dependent children expressed a preference for life-extending treatment; this was mainly due to the respondents’ desire to maximize the time they had left with their children regardless of the quality of that time [23]. Thus, treatment choice appears to be impacted by patients’ characteristics such as their age, family context, and previous therapeutic experience, and should be considered by physicians when selecting treatment. Recently, effective drugs for advanced breast cancer have been developed and the treatment options have been increasing. Therefore, determination of a treatment regimen becomes more complex, as the selection is based on various data such as predictive biomarkers that identify patients who may benefit from such therapies, clinical findings of disease (i.e., site of metastasis, time to recurrence, tumor characteristics), and patients’ treatment preferences. It is essential to communicate with patients and understand their preferences for treatment to enhance the treatment choice process and tailor treatment to an individual patient.

These study results are strengthened by the use of the patient panel of INTAGE Inc., which is a large database that encompasses a portion of the entire Japanese breast cancer patient population. Another strength of this study was the pilot phase, which allowed improvement of the questionnaire based on the respondents’ answers. Therefore, participants in the main study were able to fully comprehend the wording of the questionnaire and data credibility was maintained. The included attributes reflected the complexity of the treatment choice faced by patients and captured the most important aspects of the drivers of choice. The response options were based on the results of a clinical trial that were believable without being too extreme. However, a number of limitations also need to be listed. The small number of respondents who had experienced a relapse or metastases in this study limited generalizing the results to this patient population. Factors such as response rate or potential survival may also affect their treatment choice [24, 25]. Patients who were physically weak or had severe symptoms may not have been willing to participate in the survey, and therefore, the selection process may have created bias. The study was conducted using an online survey methodology, without any option for data collection on paper, which may have biased the study toward respondents who were confident in the use of technology and had access to it. Nevertheless, the mean age of the study population (56.7 years) is similar to the recently reported average age of 6152 Japanese women with breast cancer (i.e., 57.6 years) [26], reducing the possibility that the online format may have promoted a younger patient population. Hormone sensitivity of respondents presumed by their response to the specific questions may have been subject to reporting bias. Finally, since the choice sets comprised only limited numbers of treatment attributes, there may be other attributes that also affect patients’ preferences in clinical practice.

In conclusion, in this DCE study, postmenopausal patients with HR+ breast cancer in Japan professed a strong desire to receive treatment that can extend PFS even with the potentiality of grade 2 diarrhea. However, when frequency of loose stools increased to grade 3 severity, patients were more willing to sacrifice PFS to avoid experiencing more frequent loose stools. This suggests that it will be important to prevent and manage diarrhea with the goal of limiting it to grade 2 and below, to maintain patients’ motivation for treatments that can extend PFS. This study also showed that patients’ sociodemographic and clinical characteristics tend to affect patients’ treatment choices. These findings will help physicians to have discussions with their patients and develop strategies of treatment choice for postmenopausal HR+/HER2− advanced breast cancer treatment. It will be important to choose treatments that consider patients’ characteristics such as their age, family context, and therapeutic experience. This research provides new understanding of patients’ preferences for treatment; a key factor for enhancing the treatment choice process and tailoring treatment to the individual patient.

## Electronic supplementary material

Below is the link to the electronic supplementary material.

Supplementary material 1 (DOCX 59 KB)
