# Supplementary material for: Patients’ preferences for postmenopausal hormone receptor-positive, human epidermal growth factor receptor 2-negative advanced breast cancer treatments in Japan
Source: Breast Cancer. 2019 Apr 4;26(5):652–62. doi: 10.1007/s12282-019-00965-4 (PMC6694082; doi:10.1007/s12282-019-00965-4)
Supplement: Supplementary file 1 — Supplementary material 1 (DOCX 59 KB) [file 12282_2019_965_MOESM1_ESM.docx]

Supplementary MATERIAL

**Supplementary Table S1.** Example of a choice question^a^

| **Pattern 1** | **Treatment A** | **Treatment B** |  |
| --- | --- | --- | --- |
| The length of time before your breast cancer returns | 16 months | 9 months | |
| Incidence of diarrhea | 9 of 10 patients will experience diarrhea | 2 of 10 patients will experience diarrhea | |
| Frequency of loose stools | Increase of 3 loose stools per day more than usual | Increase of 6 loose stools per day more than usual | |
| Duration of the symptom  of diarrhea | 2 months | 2 weeks | |
| Route of drug administration | Intramuscular injection only (once every  4 weeks) | Oral administration (twice  a day) ＋ intramuscular injection (once every  4 weeks) |  |
| **Question**  Which treatment is more preferable?  **Please choose 1 option** | 🞏 | 🞏 |  |

^a^The questionnaires used in both the pilot and the main study were written in Japanese
